# A New Measure of Functional Evenness and Some of Its Properties

**DOI:** 10.1371/journal.pone.0104060

**Published:** 2014-08-08

**Authors:** Carlo Ricotta, Giovanni Bacaro, Marco Moretti

**Affiliations:** 1 Department of Environmental Biology, University of Rome ‘La Sapienza’, Rome, Italy; 2 CNR, Istituto di Ricerca per la Protezione Idrogeologica, Perugia, Italy; 3 BIOCONNET, Biodiversity and Conservation Network, Department of Life Science, University of Siena, Siena, Italy; 4 Swiss Federal Research Institute WSL, Birmensdorf, Switzerland; Stanford University, United States of America

## Abstract

Functional evenness is increasingly considered an important facet of functional diversity that sheds light on the complex relationships between community assembly and ecosystem functioning. Nonetheless, in spite of its relevant role for ecosystem functioning, only a few measures of functional evenness have been proposed. In this paper we introduce a new measure of functional evenness that reflects the regularity in the distribution of species abundances, together with the evenness in their pairwise functional dissimilarities. To show how the proposed measure works, we focus on changes in functional evenness calculated from Grime’s classification of plant strategies as competitors (C), stress-tolerators (S) and ruderals (R) along a post-fire successional gradient in temperate chestnut forests of southern Switzerland.

## Introduction

Biological diversity is a central concept in ecology, which has been extensively studied for over 50 years, with the aim of understanding the relationships between community composition and ecosystem functioning [1]–[2]. More recently, the concept of functional diversity has received considerable attention because it captures information on species functional traits, which is absent in traditional measures of species diversity.

Functional traits are morphological, physiological, and phenological attributes, which impact individual fitness via their effects on growth, reproduction and survival [3]. Therefore, measures of functional diversity tend to correlate more strongly than those of traditional species-diversity with ecosystem functioning [4]. The observed relationships between functional diversity and ecosystem functioning raise the question of how to measure functional diversity in meaningful ways [5]–[7]. For instance, when we look for a suitable numerical definition, we find that no single index adequately summarizes functional diversity. This is because the functional organization of communities cannot be assessed by a single measure but rather needs a multi-faceted approach [7]–[9]. According to [6] and [10], functional diversity can be summarized mainly by three complementary families of measures: functional richness, functional evenness, and functional divergence. Functional richness represents the volume of the functional space occupied by the community, functional evenness measures the regularity of the distribution of species abundances and dissimilarities in functional space and functional divergence quantifies how species diverge in their (abundance-weighted) distances from their center of gravity in functional space. For more details on the definition of functional richness, functional evenness, and functional divergence see [6].

Like the distinction between species richness and evenness in traditional diversity studies, taken together, these three components of functional diversity summarize distinct facets of the extent of trait differences among coexisting species that are expected to express different mechanisms of community assembly and species coexistence [11]–[12]. However, while the definition of functional richness is widely accepted among ecologists, the definitions of trait divergence and trait evenness are more controversial. For instance, in [6] the calculation of functional evenness is based on the minimum spanning tree, which links the *N* species of a given community in multidimensional functional space such that the total length of its *N*-1 branches is minimized. Then, functional evenness quantifies the regularity with which species are distributed along the tree, together with the evenness in their abundances.

One potential problem with the calculation of the minimum spanning tree from a species dissimilarity matrix is that if more species pairs have equal functional distances, the minimum spanning tree may not be unique. Also, according to [6], only the dissimilarity values that contribute to the construction of the minimum spanning tree, that is to say, to the ‘backbone’ of the pairwise species dissimilarity matrix, are used for the calculation of functional evenness, whereas the remaining portion of the dissimilarity matrix does not contribute to the calculation of functional evenness. As an alternative to this ‘minimum-subgraph measure’, in this paper we propose a new measure of functional evenness that takes into account all interspecies dissimilarities in a given community. To show how the proposed measure works we focus on changes in Grime’s [13]–[14] CSR functional classification of plant assemblages as competitors (C), stress-tolerators (S) and ruderals (R) along a secondary post-fire successional gradient.

## A New Measure of Functional Evenness

In most cases the information available for summarizing the functional organization of a given species assemblage is a vector of relative abundances of each of *N* species *p_i_*  =  (*p_1_*, *p_2_*,…, *p_N_*), together with an *N*×*T* matrix with values for *T* selected functional traits for each species [6]. As most functional diversity indices are built on pairwise functional dissimilarities between species, this latter matrix is first transformed to an *N*×*N* matrix of species functional dissimilarities. Given species relative abundance and dissimilarity data, we next define the average community uniqueness *U* as the expected dissimilarity between one individual of species *i* chosen at random from a given community and all other *j*-th species in the community:



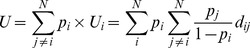
(1)


where *d_ij_* is the functional dissimilarity between species *i* and *j* (with *d_ij_*  =  *d_ji_* and *d_ii_*  =  0), the ratio 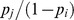 is the relative abundance of species *j* with 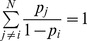, and 
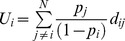
 is the expected (i.e. weighted) dissimilarity between species *i* and all other species in the community (see also [15]). This quantity may be seen as the fraction of functional characters of species *i* that is not shared with other community species. *U_i_* is therefore a measure of the ‘functional uniqueness’ of species *i*. If all species in the community are functionally similar, the functional dissimilarity *d_ij_* tends to be low and *U_i_* tends to be close to zero; on the other hand, if species *i* has unique functional characters, *d_ij_* tends to be high and hence *U_i_* tends to be high too. Average community uniqueness *U* is then obtained as the summation of the single-species uniqueness *U_i_* weighted by their relative abundances *p_i_* (see Eq. 1). If the interspecies dissimilarities *d_ij_* used for calculating *U* are in the range [0, 1] we have: 
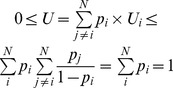
. Accordingly, since 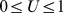, the complement of average community uniqueness 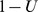 may be used for measuring the community functional redundancy [16].

Starting from average community uniqueness *U*, a straightforward measure of functional evenness consists in calculating the regularity in the distribution of single species contributions to *U*. First, the quantities 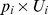 are transformed to a finite probability space by dividing each term by *U*:
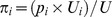
(2)


where 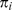 is the relative contribution of species *i* to *U* such that 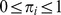 and 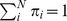. Next, like in [6], the regularity in the distribution of the quantities 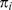 is calculated with the index of Bulla [17]:
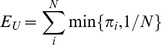
(3)


For *N* species, the index of Bulla has a minimum value not greater than 1/*N* (in case 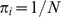
for all *i  = 1,…, N*) and a maximum value of 1. Therefore, in order to compare functional evenness between communities with a different number of species, *E_U_* must be transformed onto the unit interval. The simplest way to rescale *E_U_* between zero and one is to use the linear transformation (*E_U_* − *E_min_*)/(*E_max_* − *E_min_*), which gives a relative evenness index:
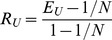
(4)


This way of calculating functional evenness is in agreement with [18], which defined functional evenness as ‘the evenness of species contribution to certain ecosystem functions within ecosystems’.

## Case Study: Vegetation Secondary Succession along a Post-Fire Gradient

### Study area

To illustrate the strengths and the weaknesses of the newly proposed measure we analyzed changes in functional evenness along a post-fire successional gradient in temperate chestnut forests of southern Switzerland [19]. The study area (11×15 km^2^) is located in Ticino near Locarno (southern Switzerland). The elevation ranges from 450 to 850 m a.s.l. along a uniform south-facing slope dominated by coppice stands of *Castanea sativa*. Due to the mild climate with wet summers and relatively dry winters, the study area is prone to fast-spreading winter surface fires of low–medium intensity that assume an important function in maintaining a mosaic of vegetation patches with different successional stages [20]. Further details on the study area are given in [21]. Vegetation was sampled at 21 sites selected according to the time elapsed since the last fire event, varying from 0 to 35 years. At each site, species abundances were visually estimated in May–June and August 1997 within 10×10 m^2^ quadrats using a seven-point ordinal scale according to [22].

### Data and Methods

We classified plant species according to Grime’s [13] theoretical triangular scheme of competitor, stress tolerator and ruderal plant strategies using fuzzy coded values such that C+S+R  =  100. Grime [13]–[14] identified two categories of external factors limiting plant performance: stress (restrictions to plant production imposed by the environment, such as shortages of water and nutrients) and disturbance (events that destroy plant production, such as herbivory, burning, or extreme climatic conditions). Therefore, based on a number of different functional traits that are diagnostic of the species adaptive strategies to different combinations of stress and disturbance, plants can be classified into competitor, stress-tolerator and ruderal strategists [23]. For most species the CSR values were assigned according to [14], while for species not occurring in the British Isles the CSR values were calculated according to the protocol in [23].

To assess community-level changes in CSR strategies along the post-fire successional gradient, we calculated the mean (abundance-weighted) values of single CSR strategies at each plot 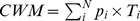, where *CWM* is the community-weighted mean value of trait *T_i_* for species *i* (i.e. the fuzzy-coded values of single C, S, and R strategies, such that 0 < *T_i_* < 100). This index is usually viewed as a measure of the dominant traits in a community and is directly related to the mass-ratio hypothesis [24], which considers the functional characters of the dominant species as the main drivers of ecosystem processes [7].

To assess changes in functional evenness along the post-fire successional gradient, we first calculated the values of *R_U_* of each plot using the Marczewski–Steinhaus coefficient of dissimilarity, which is the complement of the Ruzicka similarity index [25]–[26]. For throughout discussion on the treatment of ordinal data with metric dissimilarity measures see [27] and references therein. Next, we fitted a linear regression model for *R_U_* vs. the time since last fire. Statistical significance for the regression estimate (two-tailed test) is based on 9999 randomizations.

For the calculation of functional evenness, functionally identical species were grouped into single ‘functional species’ according to [28]. The reason for this operation will be clear in the following sections, when we discuss the properties of functional evenness; only 20 plots with at least three functional species were used in this study (see Appendix S1). The index *R_U_* was calculated with the new R function ‘FeveR’ [29] that is made available as electronic appendix to this paper (Appendix S2).

## Results

Overall, thermophilous species were preferentially associated with recently burned sites, while shade-tolerant plant species increased in unburned sites. Recently burned sites were characterized by open canopies and dense understory cover dominated by *Teucrium scorodonia*, *Rubus* sp. and *Cytisus scoparius*. Late successional stages had closer canopies dominated by *Castanea sativa*, *Prunus avium*, *Tilia cordata* and *Sorbus aria*. *Betula pendula* and *Quercus petraea* were typical subdominant tree species of unburned sites. From a functional perspective, early successional stages were typically associated to annual therophytes and geophytes with light seeds dispersed by wind and pollinated by insects that were able to exploit the longer flowering duration of ruderal species. Late successional stages were mostly characterized by perennial forest species, pollinated by wind, with short flowering duration, and with large seeds dispersed by animals [19].

**Figure 1:**
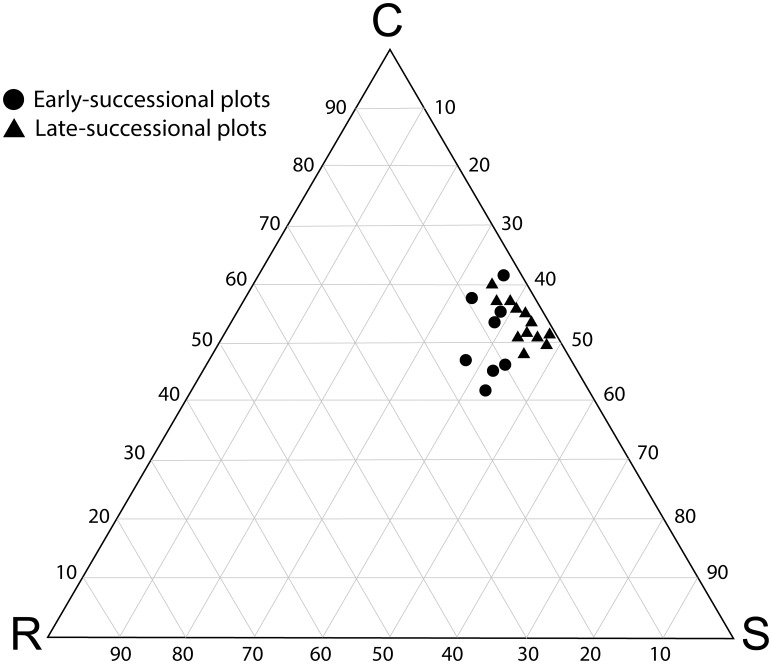
Ternary diagram of the community-weighted mean trait values of Grime’s classification of plant strategies as competitors (C), stress-tolerators (S) and ruderals (R) for 20 vegetation plots along a post-fire successional gradient. To ease the visualization of post-fire successional dynamics the plots are grouped into early- (< 10 years after fire), and late-successional stages (≥ 10 years after fire).

**Figure 2:**
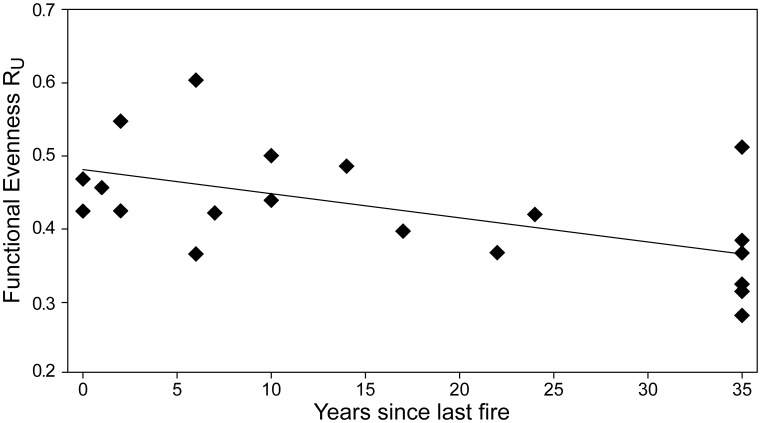
Correlation plots between functional evenness R_U_ and the time since last fire. R^2^  =  0.306; p<0.05 (two-tailed test, 9999 randomizations).

Post-fire vegetation dynamics was characterized by a significant decrease of the ruderal component along the successional gradient (Figure 1). At early successional stages (<10 years after fire), the contribution of the ruderal component to the average community functional spectrum was 9.2% (C:S:R  =  51.5%:39.3%:9.2%; mean of eight plots). At later-successional stages (≥10 years after fire), ruderal species decreased to 3.5% (C:S:R  =  53.8%:42.7%:3.5%; 12 plots). The observed decrease in the ruderal component during the post-fire vegetation recovery is associated with a moderate, though significant decrease of functional evenness with the time since last fire (R^2^  =  0.306, p < 0.05; see Figure 2).

## Discussion

The proposed evenness measure R_U_ quantifies an important aspect of the relationship between community composition and functioning. As shown by our results, changes in community composition between early-successional and late-successional stages were functionally supported. That is, along the secondary succession, the species contributions to average community uniqueness *U* became increasingly uneven.

Note that average community uniqueness is very similar to the Rao [30] quadratic diversity 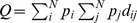
(i.e. the expected dissimilarity between two individuals chosen at random with replacement from a given community). The main difference is that in Eq. (1) expected dissimilarity is calculated imposing the restriction 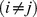, whereas in the calculation of *Q* this restriction is relaxed, such that both randomly selected individuals may belong to the same species. This is an important distinction, as for the calculation of functional evenness, it does not make sense to compare a species with itself. Note also that in accordance with the proposal of [6], we constructed our measure of functional evenness based on the index of Bulla. However, for calculating the regularity in the distribution of the quantities 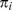, each other meaningful measure of evenness may be equally used. For review, see e.g. [31]–.

Average community uniqueness *U* offers ample flexibility in the selection of the dissimilarity measure. This is a desirable property, since in the measurement of functional diversity the issue of data type becomes a major factor. As emphasized by [34], trait matrices usually contain a mixture of nominal, ordinal, interval or ratio-scale data types [35]. Therefore, to calculate functional dissimilarities in a meaningful manner, a measure that is adequate to the statistical type(s) of selected variables should be used. Although several criteria have been proposed for choosing a suitable dissimilarity coefficient, none of them is really conclusive. As stated by Gower and Legendre [36] “a coefficient has to be considered in the context of the descriptive statistical study of which it is a part, including the nature of the data and the intended type of analysis”. In this framework, a concise ‘identification key’ for guiding the researcher in selecting an appropriate dissimilarity measure for the problem at hand can be found in [37], pp. 105–106.

Likewise, many authors have proposed a set of basic criteria that an index of evenness should meet to reasonably behave in ecological research [31]–[33], [38]. As it is usually the case in multivariate analysis, these criteria are sometimes in contrast with each other and cannot be met all at once. Among them, unlike [39] we believe that the foremost requirement for a measure of functional evenness is its independence from species richness. If richness and evenness are independent, then knowing the value of one component would put no mathematical constraints on the value of the other. For traditional evenness measures analytical methods were derived for testing their independence from species richness [39]. However, unlike traditional evenness measures that are computed solely from the relative species abundances of a given assemblage, functional evenness depends on two variables: the species dissimilarities and the frequency distribution of these species. Therefore, we empirically explored the possibility for getting the extreme evenness values of zero and one (denoting maximal unevenness and maximal evenness) irrespective of the number of species in the assemblage.

For *N* ≥ 2, *R_U_* takes its maximum value of one when all species relative abundances are perfectly evenly distributed *and* all interspecies dissimilarities 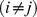 are equal to each other (denoting perfect evenness in the distribution of the *d_ij_* values, with *d_ii_*  =  0 by definition). That is, to get maximal functional evenness, we need maximal evenness in the distribution of species relative abundances *and* of interspecies dissimilarities. On the other hand, the minimum value *R_U_*  =  0 is attained when the species relative abundances *p_i_* reach maximum unevenness (i.e. one term *p_i_* approaches one, while all remaining terms *p_j_* are close to zero), irrespective of how interspecies dissimilarities are distributed. In a sense, species relative abundances are thus weighted more than interspecies dissimilarities in the calculation of *R_U_*. Although this may seem disappointing, the mass-ratio hypothesis [24] proposes that immediate controls on ecosystem functioning are mainly determined by the traits of the dominant species and are relatively insensitive to less abundant species. Hence, intuitively, if species abundances are maximally uneven, the contribution of the dominant species to ecosystem functioning is maximal, regardless of the traits of the very minor species.

In this context, a major drawback of most evenness measures, such as the index of Bulla, is that evenness does not depend continuously on the species relative abundance at *p_i_* → 0 [32], [40]. This means that a community with relative abundances (*p_i_*  =  0.5-ε, *p_j_*  =  0.5-ε, *p_k_*  =  2ε) with *ε* tending to zero has an evenness that is much less than that of community (*p_i_*  =  0.5, *p_j_*  =  0.5). This discontinuity is related to Pielou’s [41] distinction between a fully censused community and a sampled community and implies inestimability of evenness if we consider our plots as a collection of samples from a larger community. However, we agree with [42] that “the concept of a sample from a community is unrealistic, because it assumes that communities have reality as discrete units, which very few ecologists believe”. At least for sessile individuals, such as plants, it is more realistic to view our plots as fully censused pieces of biotic space at a given scale [43]. Nonetheless, those who agree with Pielou’s distinction between a fully censused and a sampled community should restrict the calculation of evenness to measures that are continuous at *p_i_* → 0 (see e.g. [32], [44]-[45].

Also, dealing with evenness measures that are calculated from species relative abundances *and* dissimilarities, such as *R_U_*, we face a similar problem at *d_ij_* → 0. For instance, a community with species relative abundances (*p_i_*  =  0.5, *p_j_*  =  0.25, *p_k_*  =  0.25) and interspecies dissimilarities (*d_ij_*  =  1, *d_ik_*  =  1, *d_jk_*  =  0) has an evenness that is much less than that of community (*p_i_*  =  0.5, *p_j_*  =  0.5; *d_ij_*  =  1). However, as species *j* and *k* are functionally identical, for the calculation of functional evenness they should be treated as they were one single entity, and there should be no difference between the evenness of the first and the second community. Therefore, to avoid problems with the presence of two or more species with identical functional traits, we introduced the additional constraint that functionally identical species are grouped into one single functional species. The meaning of this operation is that the number of species itself is not relevant to *R_U_* unless these species possess distinct functional characters [28].

To conclude, changes in functional evenness may be induced by changes in interspecies dissimilarities and/or in species abundances, as both of them contribute in shaping community functioning. Accordingly, a ‘perfect’ measure of functional evenness probably does not exist. Rather, several ‘tailored’ measures of functional evenness may be developed that result from how abundances and dissimilarities are weighted in the calculation of the index, and their specific relevance must be judged on the basis of their ability to fit the given problem of application.

## Supporting Information

Appendix S1 **Relative abundances and CSR values of the functional species used in this study.** The time since last fire (yrs) of each plot is also shown.
(DOCX)

Appendix S2 **R function ‘FeveR’ for calculating the functional evenness of a species’ assemblage.**
(DOC)
