# Supplementary material for: A New Measure of Functional Evenness and Some of Its Properties
Source: PLoS One. 2014 Aug 8;9(8):e104060. doi: 10.1371/journal.pone.0104060 (PMC4126696; doi:10.1371/journal.pone.0104060)
Supplement: Appendix S1 — Relative abundances and CSR values of the functional species used in this study. The time since last fire (yrs) of each plot is also shown. (DOCX) [file pone.0104060.s001.docx]

|  | **Relative abundances** | | | | | | | | | | | | | | | | | | | | **CSR Strategies** | | |
| --- | --- | --- | --- | --- | --- | --- | --- | --- | --- | --- | --- | --- | --- | --- | --- | --- | --- | --- | --- | --- | --- | --- | --- |
| **Functional Species** | **Plot1** | **Plot2** | **Plot3** | **Plot4** | **Plot5** | **Plot6** | **Plot7** | **Plot8** | **Plot9** | **Plot10** | **Plot11** | **Plot12** | **Plot13** | **Plot14** | **Plot15** | **Plot16** | **Plot17** | **Plot18** | **Plot19** | **Plot20** | **C** | **S** | **R** |
| **FS1** | 0.0855 | 0.0477 | 0.2516 | 0.0700 | 0.1561 | 0.1426 | 0.1402 | 0.1188 | 0.0887 | 0.0575 | 0.2041 | 0.1373 | 0.1440 | 0.1226 | 0.1200 | 0.0945 | 0.1325 | 0.1423 | 0.1124 | 0.0982 | 100 | 0 | 0 |
| **FS2** | 0.0000 | 0.0286 | 0.0943 | 0.0510 | 0.0130 | 0.0172 | 0.2258 | 0.0594 | 0.1223 | 0.1149 | 0.1002 | 0.0000 | 0.0817 | 0.0646 | 0.0000 | 0.0718 | 0.0461 | 0.1114 | 0.0253 | 0.0000 | 70 | 30 | 0 |
| **FS3** | 0.0000 | 0.0143 | 0.0000 | 0.0000 | 0.0130 | 0.0344 | 0.0282 | 0.0594 | 0.0000 | 0.0000 | 0.0000 | 0.0000 | 0.0183 | 0.0074 | 0.0000 | 0.0378 | 0.0230 | 0.0124 | 0.0253 | 0.0730 | 70 | 0 | 30 |
| **FS4** | 0.0000 | 0.0000 | 0.0000 | 0.0190 | 0.0000 | 0.0000 | 0.0000 | 0.0000 | 0.0000 | 0.0000 | 0.0000 | 0.0000 | 0.0000 | 0.0000 | 0.0000 | 0.0000 | 0.0000 | 0.0000 | 0.0000 | 0.0000 | 60 | 20 | 20 |
| **FS5** | 0.6038 | 0.6328 | 0.5629 | 0.4543 | 0.4880 | 0.6443 | 0.4186 | 0.6059 | 0.6116 | 0.6580 | 0.5937 | 0.6865 | 0.6486 | 0.6427 | 0.8185 | 0.7240 | 0.6083 | 0.4517 | 0.6966 | 0.7531 | 50 | 50 | 0 |
| **FS6** | 0.0524 | 0.0572 | 0.0000 | 0.0467 | 0.0176 | 0.0344 | 0.1025 | 0.0178 | 0.0000 | 0.0000 | 0.0649 | 0.0458 | 0.0550 | 0.0155 | 0.0000 | 0.0378 | 0.0104 | 0.0124 | 0.1124 | 0.0000 | 50 | 0 | 50 |
| **FS7** | 0.0000 | 0.0000 | 0.0000 | 0.0086 | 0.0130 | 0.0000 | 0.0000 | 0.0396 | 0.0000 | 0.0000 | 0.0000 | 0.0000 | 0.0000 | 0.0000 | 0.0000 | 0.0170 | 0.0104 | 0.0000 | 0.0000 | 0.0000 | 40 | 40 | 20 |
| **FS8** | 0.0157 | 0.0000 | 0.0629 | 0.0286 | 0.0130 | 0.0000 | 0.0000 | 0.0000 | 0.0000 | 0.0000 | 0.0000 | 0.0000 | 0.0000 | 0.0000 | 0.0000 | 0.0000 | 0.0000 | 0.0167 | 0.0000 | 0.0252 | 33.3 | 33.3 | 33.3 |
| **FS9** | 0.0000 | 0.0159 | 0.0000 | 0.0043 | 0.0000 | 0.0000 | 0.0000 | 0.0396 | 0.0306 | 0.0575 | 0.0000 | 0.0229 | 0.0092 | 0.0736 | 0.0000 | 0.0000 | 0.0230 | 0.0000 | 0.0281 | 0.0504 | 30 | 70 | 0 |
| **FS10** | 0.1204 | 0.0477 | 0.0000 | 0.0381 | 0.0000 | 0.0258 | 0.0000 | 0.0198 | 0.0000 | 0.0000 | 0.0000 | 0.0000 | 0.0000 | 0.0000 | 0.0000 | 0.0000 | 0.0230 | 0.0124 | 0.0000 | 0.0000 | 30 | 0 | 70 |
| **FS11** | 0.0873 | 0.1097 | 0.0000 | 0.0810 | 0.1236 | 0.0687 | 0.0564 | 0.0396 | 0.1193 | 0.0862 | 0.0000 | 0.0664 | 0.0183 | 0.0736 | 0.0000 | 0.0000 | 0.0565 | 0.0712 | 0.0000 | 0.0000 | 20 | 60 | 20 |
| **FS12** | 0.0000 | 0.0000 | 0.0000 | 0.0381 | 0.0000 | 0.0000 | 0.0000 | 0.0000 | 0.0000 | 0.0000 | 0.0000 | 0.0000 | 0.0000 | 0.0000 | 0.0000 | 0.0000 | 0.0000 | 0.0000 | 0.0000 | 0.0000 | 20 | 40 | 40 |
| **FS13** | 0.0000 | 0.0000 | 0.0000 | 0.0129 | 0.0000 | 0.0000 | 0.0000 | 0.0000 | 0.0000 | 0.0000 | 0.0000 | 0.0000 | 0.0000 | 0.0000 | 0.0000 | 0.0000 | 0.0000 | 0.0000 | 0.0000 | 0.0000 | 20 | 20 | 60 |
| **FS14** | 0.0000 | 0.0318 | 0.0283 | 0.0857 | 0.0325 | 0.0172 | 0.0000 | 0.0000 | 0.0275 | 0.0259 | 0.0000 | 0.0412 | 0.0248 | 0.0000 | 0.0615 | 0.0170 | 0.0565 | 0.1077 | 0.0000 | 0.0000 | 0 | 100 | 0 |
| **FS15** | 0.0000 | 0.0000 | 0.0000 | 0.0000 | 0.0130 | 0.0000 | 0.0000 | 0.0000 | 0.0000 | 0.0000 | 0.0000 | 0.0000 | 0.0000 | 0.0000 | 0.0000 | 0.0000 | 0.0000 | 0.0371 | 0.0000 | 0.0000 | 0 | 70 | 30 |
| **FS16** | 0.0349 | 0.0143 | 0.0000 | 0.0381 | 0.1171 | 0.0000 | 0.0282 | 0.0000 | 0.0000 | 0.0000 | 0.0371 | 0.0000 | 0.0000 | 0.0000 | 0.0000 | 0.0000 | 0.0104 | 0.0248 | 0.0000 | 0.0000 | 0 | 50 | 50 |
| **FS17** | 0.0000 | 0.0000 | 0.0000 | 0.0238 | 0.0000 | 0.0155 | 0.0000 | 0.0000 | 0.0000 | 0.0000 | 0.0000 | 0.0000 | 0.0000 | 0.0000 | 0.0000 | 0.0000 | 0.0000 | 0.0000 | 0.0000 | 0.0000 | 0 | 0 | 100 |
| **Time since last fire (Yrs)** | 0 | 0 | 1 | 2 | 2 | 6 | 6 | 7 | 10 | 10 | 14 | 17 | 22 | 24 | 35 | 35 | 35 | 35 | 35 | 35 |  |  |  |

**Appendix S1** Relative abundances and CSR values of the functional species used in this study. The time since last fire (yrs) of each plot is also shown.
