# Supplementary material for: A New Measure of Functional Evenness and Some of Its Properties
Source: PLoS One. 2014 Aug 8;9(8):e104060. doi: 10.1371/journal.pone.0104060 (PMC4126696; doi:10.1371/journal.pone.0104060)
Supplement: Appendix S2 — R function ‘FeveR’ for calculating the functional evenness of a species’ assemblage. (DOC) [file pone.0104060.s002.doc]

**Appendix S2**

R function ‘FeveR’ for calculating the functional evenness of a species’ assemblage. This program is free software: you can redistribute it and/or modify it under the terms of the GNU. General Public License <http://www.gnu.org/licenses/>.

**Disclaimer:** users of this code are cautioned that, while due care has been taken and it is believed accurate, it has not been rigorously tested and its use and results are solely the responsibilities of the user.

**Description**: given a matrix of N species (absolute or relative) abundances × M plots, together with an N×N matrix of functional distances between species, this function calculates the functional evenness of each plot as described in the main text. For the calculation of functional evenness, the species absolute abundances in the input matrix are first transformed to species' relative abundances within each plot.

**Dependencies**: None

**Usage**: FeveR(plot_matrix, functional_distance_matrix)

**Arguments**

*Abundances:* a matrix of N species × M plots containing the (absolute or relative) abundances of all species in the functional distance matrix. Rows are plots and columns are species.

*Distances:* an N×N matrix of species’ functional distances or an object of class ‘dist’ [obtained by functions like vegdist in package vegan (Oksanen et al. 2013), or gowdis in package FD (Laliberté and Shipley 2011)].

*Output:* the function returns a vector with the index values calculated as described in the main text.

**Function Syntax**

FeveR<-function(abundances, distances)

{

rel_abundance<-sweep(abundances, 1, rowSums(abundances), "/")

rel_abu_matrix<-as.matrix(rel_abundance)

n_plot<-nrow(rel_abu_matrix)

n_species<-ncol(rel_abu_matrix)

dista_matrix<-as.matrix(distances)

###BULLA1

index_array<-rep(NA, n_plot)

for(i in 1:n_plot)

{mat_develop<-rep(rel_abu_matrix[i,],n_species)

matrix_two<-(t(matrix(mat_develop, nrow=n_species,ncol=n_species)))

matrix_non<-matrix(mat_develop, nrow=n_species,ncol=n_species)

uni<-matrix(1,nrow=n_species,ncol=n_species)

subtraction<- uni-matrix_non

division<-matrix_two/subtraction

moltiplication<-division*dista_matrix

row_sums<-rowSums(moltiplication)

per_abundance<-row_sums*rel_abu_matrix[i,]

great_sum<-sum(per_abundance)

divised<-per_abundance/great_sum

espress<-which(per_abundance>0)

S<-length(espress)

bulla<-rep(NA, length(divised))

for(l in 1:length(divised))

{

bulla[l]<-min(divised[l], 1/S)

}

indice<-sum(bulla)

norm_index<-(indice-(1/S))/(1-(1/S))

index_array[i]<-norm_index

}

return(index_array)

}

**References**

Laliberté E, Shipley B. 2011. FD: measuring functional diversity from multiple traits, and other tools for functional ecology. R package version 1.0-11. <http://cran.r-project.org/web/packages/FD/>

Oksanen J, Blanchet FG, Kindt R, Legendre P, Minchin PR, O'Hara RB, Simpson GL, Solymos P, Stevens MHH, Wagner H 2013. Vegan: Community Ecology Package. R package version 2.0-10. <http://cran.r-project.org/package/vegan>
